# Neuroimaging evidence of glymphatic system dysfunction in possible REM sleep behavior disorder and Parkinson’s disease

**DOI:** 10.1038/s41531-022-00316-9

**Published:** 2022-04-29

**Authors:** Xiaoli Si, Tao Guo, Zhiyun Wang, Yi Fang, Luyan Gu, Lanxiao Cao, Wenyi Yang, Ting Gao, Zhe Song, Jun Tian, Xinzhen Yin, Xiaojun Guan, Cheng Zhou, Jingjing Wu, Xueqin Bai, Xiaocao Liu, Guohua Zhao, Minming Zhang, Jiali Pu, Baorong Zhang

**Affiliations:** 1 Department of Neurology, Second Affiliated Hospital, School of Medicine, Zhejiang University, 310009 Hangzhou, Zhejiang China; 2 Department of Neurology, Fourth Affiliated Hospital, School of Medicine, Zhejiang University, N1 Avenue, 322000 Yiwu, Zhejiang China; 3 Department of Radiology, Second Affiliated Hospital, School of Medicine, Zhejiang University, 310009 Hangzhou, Zhejiang China

**Keywords:** Parkinson's disease, Parkinson's disease

## Abstract

Alpha-synucleinopathy is postulated to be central to both idiopathic rapid eye movement sleep behaviour disorder (iRBD) and Parkinson’s disease (PD). Growing evidence suggests an association between the diminished clearance of α-synuclein and glymphatic system dysfunction. However, evidence accumulating primarily based on clinical data to support glymphatic system dysfunction in patients with iRBD and PD is currently insufficient. This study aimed to use diffusion tensor image analysis along the perivascular space (DTI-ALPS) to evaluate glymphatic system activity and its relationship to clinical scores of disease severity in patients with possible iRBD (piRBDs) and those with PD. Further, we validated the correlation between the ALPS index and the prognosis of PD longitudinally. Overall, 168 patients with PD, 119 piRBDs, and 129 healthy controls were enroled. Among them, 50 patients with PD had been longitudinally reexamined. Patients with PD exhibited a lower ALPS index than those with piRBDs (*P* = 0.036), and both patient groups showed a lower ALPS index than healthy controls (*P* < 0.001 and *P* = 0.001). The ALPS index and elevated disease severity were negatively correlated in the piRBD and PD subgroups. Moreover, the ALPS index was correlated with cognitive decline in patients with PD in the longitudinal analyses. In conclusion, DTI-ALPS provided neuroimaging evidence of glymphatic system dysfunction in piRBDs and patients with PD; however, the potential of assessing the pathological progress of α-synucleinopathies as an indicator is worth verifying. Further development of imaging methods for glymphatic system function is also warranted.

## Introduction

Parkinson’s disease (PD) is a progressive neurodegenerative disease characterised by the loss of dopaminergic neurons in the substantia nigra and the presence of α-synuclein (α-syn) aggregations, called Lewy bodies, in the surviving neurons^1^. One of the best-characterised symptoms of prodromal PD is idiopathic rapid eye movement sleep behaviour disorder (iRBD), which is an early-stage α-synucleinopathy caused by α-syn deposition in the sublaterodorsal tegmental nucleus^2^. An imbalance between the production and clearance of α-syn and its consequent accumulation plays a pivotal role in the pathogenesis of PD^3,4^. However, the clearance mechanism of α-syn has been poorly studied.

The brain lymphatic drainage system, including a brain-wide network of perivascular spaces (PVSs), termed the ‘glymphatic system’, may contribute to the rapid clearance of macromolecules and protein aggregation in cerebrospinal fluid (CSF) circulation, including amyloid-β (Aβ) and tau^5–8^. Impairment of glymphatic waste clearance is involved in the development of several neurodegenerative disorders such as Alzheimer’s disease^9,10^. However, studies assessing this system in the field of PD are scarce. Recently, Zou et al. blocked the meningeal lymphatic vessels in A53T mice and observed α-syn deposition 6 weeks later, along with motor dysfunction, strongly suggesting that glymphatic system dysfunction aggravates the accumulation of α-syn and further accelerates the disease progression of PD^11^. However, to date, there is limited evidence supporting the association between glymphatic system malfunction and α-synucleinopathy in humans. Therefore, identifying a neuroimaging marker to detect glymphatic system changes in patients with iRBD and PD is challenging and crucial.

Glymphatic magnetic resonance imaging (MRI) [intrathecal injections of gadolinium-based contrast agents] has been used in humans to visualise the glymphatic system^12,13^. However, broad administration of gadolinium-based contrast agents is unrealistic, and at doses >1.0 mmol/kg, severe neurotoxic complications may occur^14^. Therefore, the development of a noninvasive measure is required. Recently, a technique called diffusion tensor image analysis along the perivascular space (DTI-ALPS), which was introduced to assess glymphatic function without the need for a contrast agent injection has been demonstrated using classical glymphatic MRI^15^ and validated in several neurodegenerative disorders^16–18^. Here, we utilised DTI-ALPS to compare glymphatic activity in patients with PD, patients with possible iRBD (piRBDs), and healthy controls (HCs). Then, in a follow-up study, we assessed the correlation between the ALPS index and the progression of PD. Finally, we hypothesised that the glymphatic system might be impaired in prodromal and clinical PD and correlated with the pathogenesis of α-synucleinopathy.

## Results

### Demographics and clinical data

In total, 168 patients with PD, 119 piRBDs and 129 HCs were included. The demographic and clinical data are shown in Table 1. Between the three groups, there was a significant effect on education, smoking, hypertension, hyperlipidaemia and scores of MMSE, MoCA, HAMD, HAMA, RBD-HK (II) and UPDRS III (*P* < 0.05). Post hoc tests revealed that education duration (piRBD < PD < HC), smoking (HC < piRBD, PD < piRBD), hypertension (PD < piRBD), MMSE and MoCA scores (piRBD < PD < HC), HAMD and HAMA scores (piRBD > HC, PD > HC), RBD-HK (II) score (HC < PD < piRBD), and UPDRS III score (HC < piRBD < PD) were significantly different across the groups (*P* < 0.05).

**Table 1:** Baseline characteristics and Imaging findings of the three participant groups.

	HC (*n* = 129)	piRBD (*n* = 119)	PD (*n* = 168)	*P* value	Post hoc tests *P* value		
				HC vs piRBD vs PD	HC vs piRBD	HC vs PD	piRBD vs PD
Clinical variables							
Age, years	61.96 ± 7.21	61.90 ± 7.57	59.85 ± 9.88	0.138	–	–	–
Sex (F/M)	70/59	57/62	72/96	0.149	–	–	–
Education, years	9.63 ± 3.63	6.36 ± 3.86	8.31 ± 4.71	0.000**	0.000**	0.011**	0.001**
Smoking	32/97	51/68	40/128	0.001**	0.003**	0.843	0.001**
Hypertension	45/84	48/71	42/126	0.019*	0.376	0.064	0.006**
Hyperlipidemia	18/111	8/111	7/161	0.007**	0.063	0.003**	0.338
Hyperglycaemia	7/122	12/107	20/148	0.157	–	–	–
MMSE	28.26 ± 1.80	25.61 ± 4.12	26.80 ± 3.83	0.000**	0.000**	0.003**	0.003**
MoCA	24.54 ± 3.61	20.21 ± 5.31	22.08 ± 5.59	0.000**	0.000**	0.001**	0.001**
HAMD	2.43 ± 2.89	6.26 ± 5.11	6.06 ± 5.14	0.000**	0.000**	0.000**	1.000
HAMA	2.88 ± 3.55	7.22 ± 5.80	5.50 ± 4.80	0.000**	0.000**	0.000**	0.115
RBDQ-HK total	8.46 ± 6.25	33.08 ± 12.21	16.47 ± 13.34^#^	0.000**	0.000**	0.000**	0.000**
RBDQ-HK II	1.99 ± 3.15	18.24 ± 10.06	8.02 ± 9.45^#^	0.000**	0.000**	0.000**	0.000**
ESS	6.13 ± 4.74	NA	5.93 ± 5.07	NA	NA	0.518	NA
PDSS	129.54 ± 17.29	NA	125.64 ± 20.64	NA	NA	0.151	NA
UPDRS III	0.58 ± 1.26	5.36 ± 7.49	21.64 ± 12.37	0.000**	0.000**	0.000**	0.000**
Imaging findings							
ALPS index	1.31 ± 0.17	1.25 ± 0.17	1.20 ± 0.17	0.000**	0.001*	0.000**	0.036*

*HC* healthy control, *iRBD* idiopathic rapid eye movement sleep behaviour disorder, *PD* Parkinson’s disease, *F* female, *M* male, *MMSE* Mini-mental State Examination, *MoCA* Montreal Cognitive Assessment, *HAMD* Hamilton Depression Scale, *HAMA* Hamilton Anxiety Scale, *RBDQ-HK* REM Sleep Behaviour Disorder Questionnaire-Hong Kong, *ESS* Epworth Sleepiness Scale, *PDSS* The Parkinson’s Disease Sleep Scale, *NA* not available, *ALPS* analysis along the perivascular space.

^#^Data from 88 PD patients assessed by RBDQ-HK scale.

*Significant result with *P* < 0.05.

**Significant result with *P* < 0.05; – not significant after correction of multiple comparisons, *P* > 0.05.

### ALPS-index analyses

**Fig. 1:**
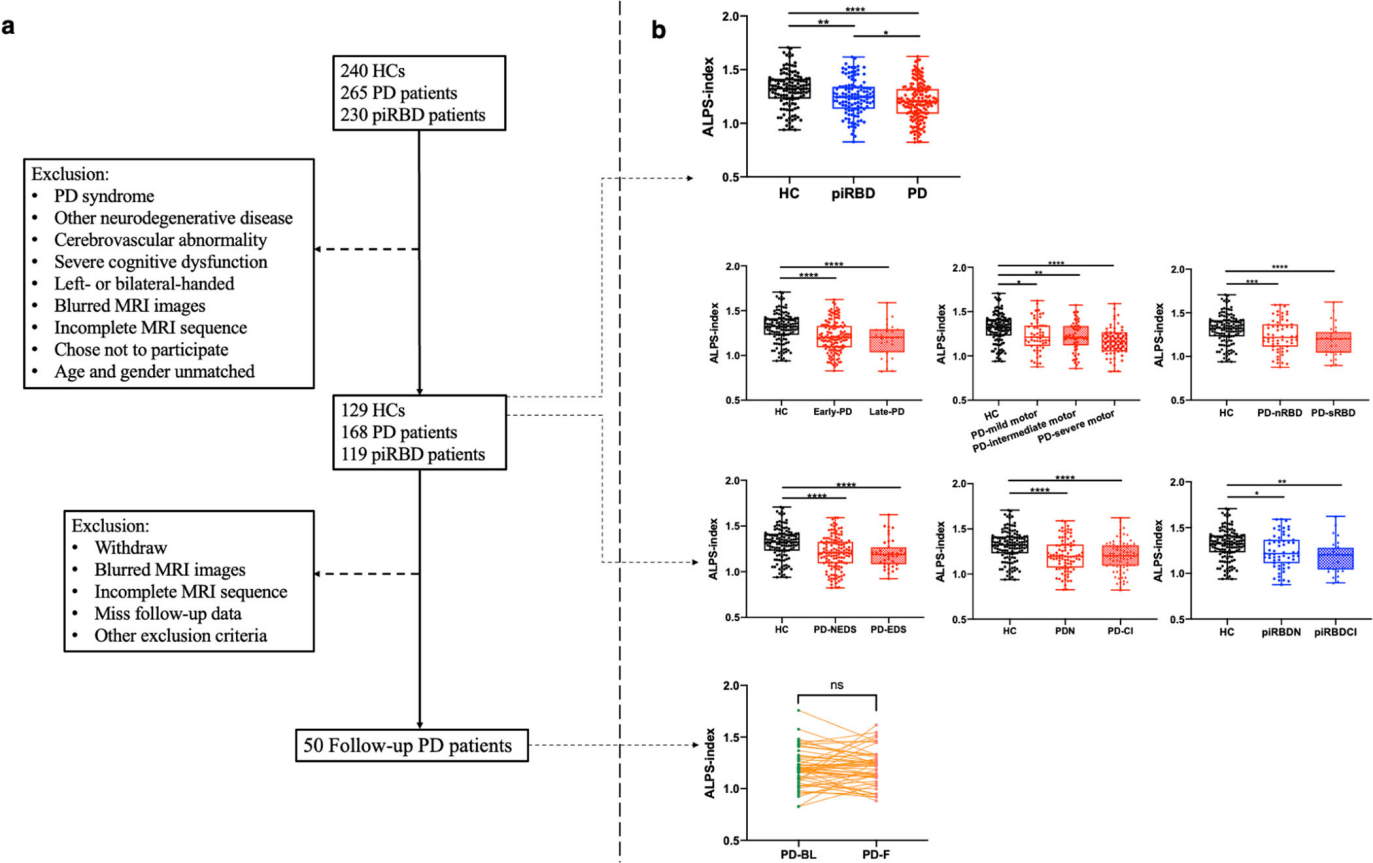
Subject screening workflow and quantitative analysis of the ALPS index. **a** The subject screening workflow. **b** Data are presented as box plots, with the box representing the median and the two middle quartiles (25–75%). Whiskers are the maxima and minima. Different pattern of ALPS index among three groups (PD < piRBD < HC, *P* < 0.001). Post hoc test revealed a higher ALPS index in the HC group than in the piRBD (*P* = 0.001) and PD groups (*P* < 0.001), and a higher ALPS index in the piRBD group than in the PD group (*P* = 0.036). Subgroup analysis of PD or piRBD patients revealed no significant difference (*P* > 0.05). Longitudinal ALPS index between PD-BL and PD-F revealed no significant difference (p > 0.05).

ICCs revealed a strong agreement between the two raters. Average ICCs for the ALPS index was 0.877 [confidence interval (CI): 0.856–0.896, *P* < 0.001]. The ALPS index of the three groups is summarised in Table 1 and Fig. 1b. There were significant differences in the ALPS index across three groups (*P* < 0.001). Post hoc tests revealed a higher ALPS index in the HC group than in the piRBD (*P* = 0.001) and PD groups (*P* < 0.001), and a higher ALPS index in the piRBD group than in the PD group (*P* = 0.036). The ALPS index was reduced in piRBDN compared with that in HCs but was higher than that in the PD group (*P* < 0.05) (Supplementary Table 1). Moreover, in the multivariable analysis, age, cognition, mood, and ALPS index (OR = 0.014; 95% CI: 0.002–0.075, *P* < 0.001) were associated with the discrimination of PD (Supplementary Table 2). This model achieved an area under curve of 0.832 (95% CI: 0.786–0.878, *P* < 0.001) (Supplementary Fig. 1). There was no significant difference in the ALPS index between healthy males and females (*P* = 0.063). The subgroup analysis revealed no significant differences between the piRBD-CI and piRBDN (Supplementary Table 1), early-PD and late-PD, PD-CI and PDN, and PD-sRBD and PD-nRBD or PD motor subgroups (*P* > 0.05) (Fig. 1b).

### Relationships between the ALPS index and clinical disease severity

**Fig. 2:**
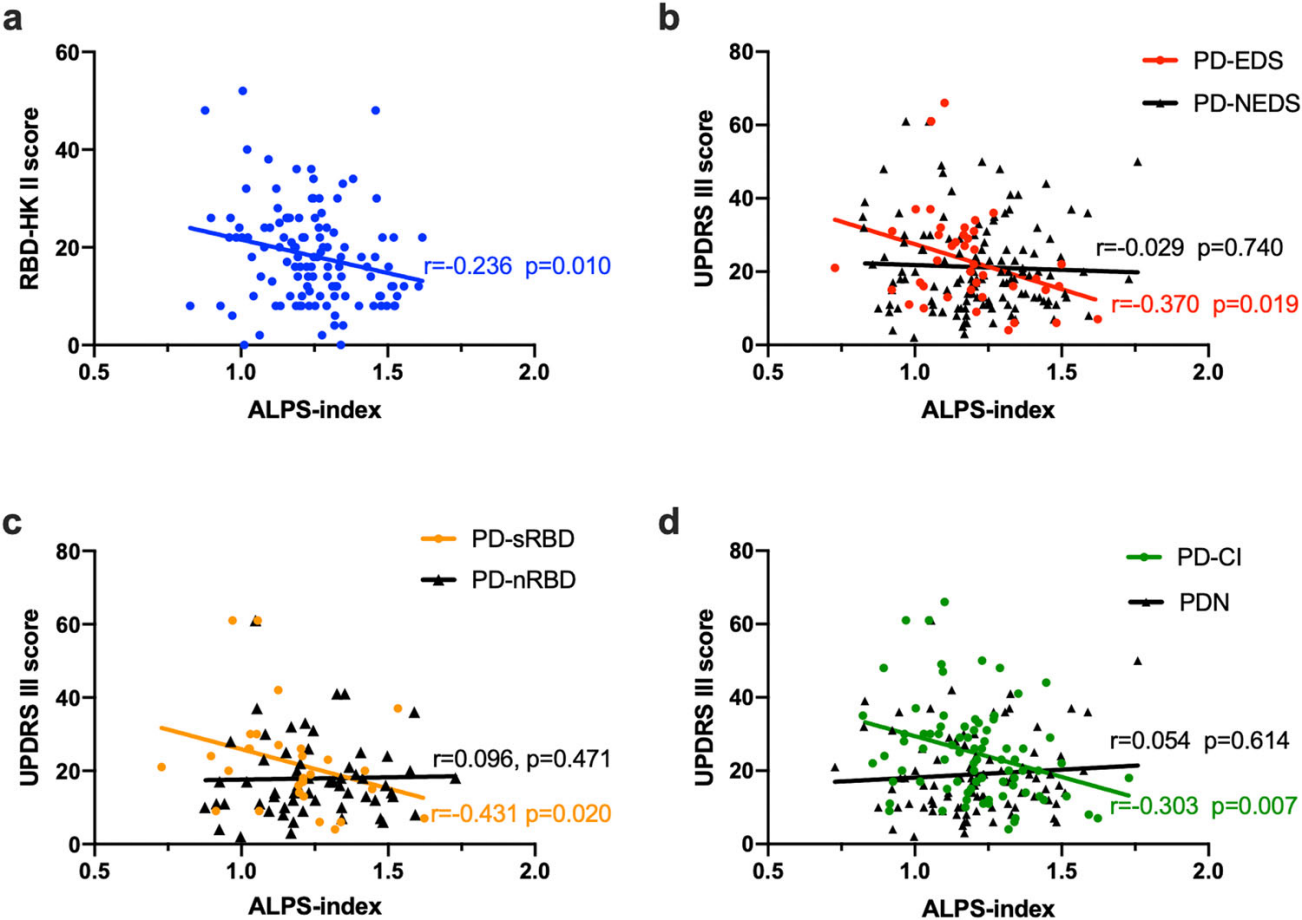
Correlation between ALPS index and clinical features. Spearman correlation revealed the ALPS index is negatively correlated with the RBDQ-HK II score in piRBD group (*r* = −0.236, *P* = 0.010, corrected *P* = 0.045) (**a**), the UPDRS III score in PD-EDS group (*r* = −0.370, *P* = 0.019, corrected *P* = 0.045) (**b**), PD-sRBD group (*r* = −0.431, *P* = 0.020, corrected *P* = 0.045) (**c**) and PD-CI group (*r* = −0.303, *P* = 0.007, corrected *P* = 0.045) (**d**).

Spearman’s correlation analysis showed a significant negatively correlation between the ALPS index and RBDQ-HK II score in the piRBD group (*r* = −0.236, *P* = 0.010, corrected *P* = 0.045) (Fig. 2a). In the PD group, there was no significant correlation between the ALPS index with UPDRS III or MoCA scores (*P* > 0.05). There were significant negatively correlations between the ALPS index and UPDRS III scores in the PD-EDS (*r* = −0.370, *P* = 0.019, corrected *P* = 0.045), PD-sRBD (*r* = −0.431, *P* = 0.020, corrected *P* = 0.045), and PD-CI (*r* = −0.303, *P* = 0.007, corrected *P* = 0.045) groups, respectively (Fig. 2b–d) (Supplementary Table 3).

### Longitudinal DTI-ALPS analysis

**Fig. 3:**
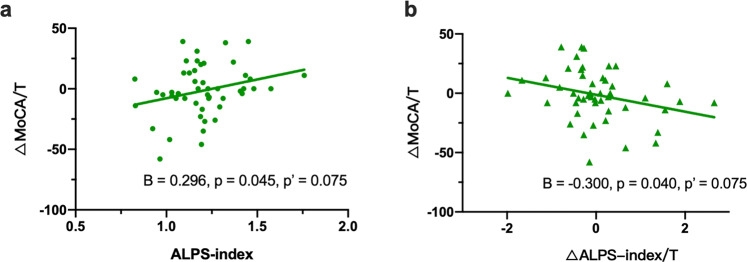
Correlation between ALPS index and the cognitive progress. In the linear regression model, there was a positive correlation between the baseline ALPS-index value and the ΔMoCA/T score (B = 0.296, *P* = 0.045, *P*’= 0.075) (**a**) and a negative correlation between the ΔALPS index/T and the ΔMoCA/T score (B = −0.300, *P* = 0.040, *P*’ = 0.075) (**b**). *P*’, corrected *P*.

In the follow-up study, there was no significant difference in the ALPS-index value between patients with PD-BL and those with PD-F (Table 2 and Fig. 1b). In the linear regression model with age and sex as covariates, ΔALPS index/T was negatively correlated with the baseline ALPS index (B = −0.496, *P* < 0.001, corrected *P* < 0.001). Furthermore, age, sex, and LED, were included as covariates, and there was no correlation between ΔUPDRS III/T and the baseline ALPS index (B = 0.115, *P* = 0.442, corrected *P* = 0.442) or ΔALPS index/T (B = 0.146, *P* = 0.332, corrected *P* = 0.415). After including age, sex, education, and LED as covariates, we found that ΔMoCA/T was positively correlated with baseline ALPS index (B = 0.296, *P* = 0.045, corrected *P* = 0.075), and negatively correlated with ΔALPS index/T (B = −0.300, *P* = 0.040, corrected *P* = 0.075) (Fig. 3).

**Table 2:** Demographics of the follow-up participant groups.

	PD-BL	PD-F	*P* value
Age, years	60.27 ± 8.16	62.34 ± 8.09	0.000**
Smoking	13/37	14/36	0.822
Hypertension	19/31	21/29	0.683
Hyperlipidemia	3/47	3/47	1.000
Hyperglycaemia	8/42	4/46	0.218
MMSE	27.20 ± 3.57	27.14 ± 3.04	0.696
MoCA	22.68 ± 5.34	22.44 ± 4.98	0.539
HAMD	6.22 ± 4.58	6.60 ± 0.04	0.935
HAMA	5.52 ± 4.15	7.12 ± 5.23	0.021*
ESS	5.78 ± 4.73	6.64 ± 5.07	0.281
PDSS	127.42 ± 21.81	121.38 ± 19.68	0.021*
Disease duration, years	3.50 ± 2.99	5.26 ± 2.79	0.000**
UPDRS III	22.96 ± 13.67	20.98 ± 13.36	0.144
H–Y stage	2.34 (2.38–2.50)	2.17 (2.00–2.50)	0.0503
LED	305.70 ± 286.60	543.58 ± 313.97	0.000**

*PD-BL* PD at baseline, *PD-F* PD follow-up, *MMSE* Mini-mental State Examination, *MoCA* Montreal Cognitive Assessment, *HAMD* Hamilton Depression Scale, *HAMA* Hamilton Anxiety Scale, *ESS* Epworth Sleepiness Scale, *PDSS* The Parkinson’s Disease Sleep Scale, *UPDRS* Unified Parkinson’s Disease Rating Scale, *H–Y stage* Hoehn–Yahr scale.

*Significant result with *P* < 0.05, **significant result with *P* < 0.01.

## Discussion

This cohort study of piRBDs and patients with PD found that the ALPS-index pattern might represent a possible prodromal and clinical indicator of PD. First, we found that patients with PD exhibited a lower ALPS index than that of piRBDs, and both groups showed a lower ALPS index than that of HCs (PD < piRBD < HC). Moreover, a lower ALPS index was associated with elevated disease severity in both the piRBD and PD subgroups. Finally, we demonstrated the potential of the ALPS index as an imaging marker to predict cognitive decline in patients with PD. Taken together, we provided neuroimaging evidence of glymphatic system dysfunction in piRBDs and patients with PD in vivo and discovered that the ALPS index might be a cardinal marker of characteristic brain glymphatic system alterations across prodromal and clinical stages of PD.

**Fig. 4:**
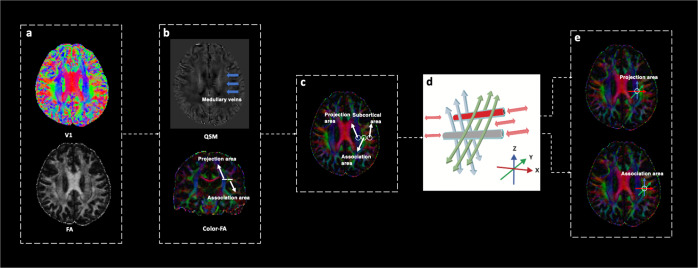
Schematic drawing of the diffusivity measurement using the DTI-ALPS method. **a** Steps to get the colour-coded FA map. **b** Select a slice in which the direction of medullary veins runs perpendicular to the ventricle wall. **c** Colour-coded FA map showing the ROI regions of the projection fibres (blue; *z* axis), association fibres (green; *y* axis), and the subcortical fibres (red; *x* axis). **d** Schematic indicating the relationship between the direction of the PVS (grey cylinders) and the fibres (the direction of the PVS is perpendicular to both projection and association fibres). **e** Placing two ROIs in the area of the projection fibres (blue) and the area of the association fibres (green).

In the glymphatic system, CSF and interstitial fluid (ISF) interchange through an influx of CSF along the loose fibrous matrix of periarterial spaces and convective ISF flux within the tissue toward the perivenous space surrounding the deep veins^19^. In the periventricular region, which is abundant with medullary arteries and veins, the PVS run in the right–left direction (*x* axis) and are the major drainage pathways of the glymphatic system. By measuring the diffusivity of those vertical projection fibres and association fibres from the compartment along the direction of the PVS (Fig. 4d), we can calculate the ALPS index, which represents the water diffusivity of the glymphatic system on the slice^17^. Recently, Zhang et al. indicated that the ALPS index was significantly related to the glymphatic clearance function calculated on classical glymphatic MRI, representing the glymphatic clearance function. Here, we demonstrated the DTI-ALPS method, which yielded results within several minutes and had good stability and intra-observer agreement. We suggested that in the future, this method might be useful for monitoring the status of the glymphatic system in clinical settings.

**Fig. 5:**
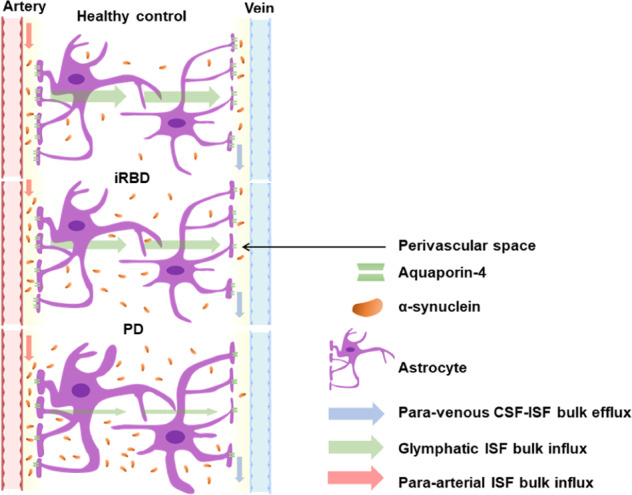
A proposed possible mechanism underlying the α-syn deposition and glymphatic system. The glymphatic system is a waste drainage system that is driven by periarterial ISF bulk influx (red arrow), glymphatic ISF bulk influx (blue arrow), and perivenous CSF-ISF efflux (green arrow). In prodromal (iRBD) and the early stage of PD, the deposition of α-syn may reduce glymphatic clearance and subsequently lead to the progressive aggregation of α-syn, which further exacerbates a vicious cycle described above and leads to dopaminergic neuronal degeneration and symptom malfunction.

In this study, the ALPS index was reduced in piRBDs compared to HCs and is further reduced in PDs compared to piRBDs (PD < piRBD <HC); this suggests that the glymphatic system might have been impaired since the prodromal phase of PD and that the disease progressed from the prodromal to the clinical stage. A previous study indicated that prolonged exposure to Aβ in CSF suppressed glymphatic transport before the presence of substantial Aβ deposition^9^. Although most α-syn is intracellular, it also spreads extracellularly^20,21^ and may induce microenvironmental responses^22^. Aquaporin-4 (AQP4) has been proposed to support PVS fluid and solute movement along the glymphatic system^23^. In A53T mice, glymphatic drainage was reduced, accompanied by impaired polarisation of AQP4 closely surrounding α-syn deposition^11^. A previous study reported delayed lymphatic drainage in mice injected with preformed α-syn fibrils and revealed that the lymphatic endothelial barrier dysfunction was induced by α-syn-laden meningeal macrophages^24^. Recent studies have proven that the PVS system is the site of antigen presentation^25^ and that it may be a hotspot of synucleinopathies^26^. These experiments are consistent with our findings that glymphatic dysfunction might be closely related to α-syn accumulation. Furthermore, we hypothesise that the glymphatic dysfunction was α-syn-related since the early stage of α-synucleinopathy and that its malfunction caused increased α-syn deposition, leading to a vicious circle (Fig. 5). Besides, other pathologies such as neuroinflammation^27^, reactive astroglioses^28^ and sleep abnormalities^29^ may also induce glymphatic dysfunction, and further animal and human studies are required to elucidate these processes.

We observed a negative correlation between the ALPS index and RBDQ-HK II score in the piRBD group and a negative correlation between the ALPS index and UPDRS III score in the PD-sRBD and PD-EDS groups (Fig. 2a–c). These associations are presumably attributable to the reduced efficacy of the glymphatic system as a result of sleep disturbance^30,31^, as the function of the glymphatic fluid is enhanced by sleep and regulated by circadian rhythms^32^. Previous studies revealed slow-wave sleep is the stage at which glymphatic drainage is enhanced^29^. A recent clinical study indicated that increased brain parenchyma mean water diffusivity was positively related to the proportion of total sleep time spent in the REM phase in healthy people^33^. As iRBD patients complained about the disruption of REM sleep due to the RBD symptoms commonly and changes in EEG signature of non-REM sleep have been observed in iRBD^34^, and a potential role of REM sleep oscillations in neurovascular coupling possibly affecting glymphatic activity^35,36^, we considered iRBD might result in decreased glymphatic function. Furthermore, several clinical studies have shown that patients with iRBD also had circadian rhythm disturbances, as evidenced by altered gene expression, rest–activity patterns and delayed melatonin secretion^37,38^. Therefore, it is reasonable to assume that RBD possibly affects glymphatic capacity. EDS is a behavioural biomarker of circadian rhythm disorder and a common non-motor (NM) symptom in PD^39,40^. Consistent with previous studies, our results suggest that the disease severity of patients with PD-EDS is closely related to the glymphatic system. A previous postmortem study showed that healthy elderly individuals with fragmented sleep had more α-syn aggregation in the substantia nigra^41^. Taken together, these findings indicate that there may be an association between sleep, the glymphatic system and α-synucleinopathy.

Consistent with a previous study^18^, a lower ALPS index in this study was associated with higher UPDRS III scores in the PD-CI group (Fig. 2d), and the baseline ALPS index was positively associated with the ΔMoCA/T (Fig. 3). Cognitive impairment is one of the most important NM symptoms of PD, and its incidence significantly increases with increasing disease duration^42^, as well as with significant α-syn, Aβ and tau protein deposition in the brain parenchyma^43–45^. Furthermore, previous autopsy and cytology studies have demonstrated that Aβ, tau and α-syn could mutually promote deposition^46–48^, and that reduced Aβ clearance likely promotes α-syn deposition and aggravates the pathological progression of PD. Given that the glymphatic system is an important pathway for protein deposition^11,49^, we speculated that glymphatic dysfunction might lead to increased Aβ and α-syn deposition and that improved glymphatic system function helped decelerate or even reversed the progression of cognitive impairment in PD. These findings provide a new perspective for future research. Future investigations on the glymphatic system and its role in the relationship between NM features and PD are warranted.

We did not find any significant difference among PD subgroups with respect to the ALPS index. Further, we found no correlation among the ALPS index, clinical symptom severity, and motor progression in the PD group. We consider the following possible explanations for these observations. First, most patients with PD were in the early stage (H–Y ≤ 2.5), which may account for the statistically insignificant difference. Besides, the ALPS index was designed to measure the diffusion in the periventricular area only, and it may not be sensitive enough to detect global glymphatic drainage differences between PD subgroups. In this study, the ΔALPS index/T was negatively correlated with the baseline ALPS index. However, no study has yet monitored the progression of the glymphatic system in PD. Moreover, the sample size in the follow-up study was relatively small, and the follow-up period was short and had large variation, which may have led to insufficient validity with respect to the statistical findings. In the future, a multi-centre study with a longitudinal cohort should be performed in this regard. Furthermore, experimental research is necessary to monitor the progression of glymphatic function in PD.

The major limitation of this study was the diagnostic issue for the piRBD group. Although RBDQ-HK has high internal consistency and test–retest reliability to screen piRBDs in China (sensitivity = 82.2%, specificity = 86.9%, positive predictive value = 86.3%, and negative predictive value = 83.0%^50,51^), considering the absence of comprehensive neuropsychological tests and the substantial difference in MoCA scores, especially in patients with piRBD, early-stage dementia with Lewy bodies without parkinsonism may be included in the piRBD group. In the future, we plan to follow-up on these piRBDs in a longitudinal cohort study to further corroborate the current findings. Regarding gender limitation, though there is no significant difference in the ALPS index between males and females in the HC group, there might be residual problems after PSM, which is an imperfect technique. Since DTI-ALPS is an innovative measure, it is important to validate potential differences according to sex. Second, we did not take other pathological markers into account. We plan to investigate the associations between pathological biomarkers, glymphatic system and clinical status in PD to better understand the underlying pathology in the future. Moreover, this is a single-centre study with limited geographic and demographic variability. Since there is a large overlap in the ALPS index of PD and HC and some variation that could not capture longitudinal changes, ALPS-index potential as an index to assess the pathological progression of α-synucleinopathies is worth validating. However, this study could be considered valid preliminary research, and our results deserve to be replicated with a multi-centre study. Finally, due to the observational nature of our study, which lacks an intervention to modify the glymphatic system, further experimental evidence is needed to confirm that DTI-ALPS measures glymphatic function.

In conclusion, there was a sequential decrease in the ALPS index from prodromal PD to clinical PD, and the ALPS index was related to clinical measures of disease severity in piRBDs and patients with PD. In addition, DTI-ALPS might be a potential imaging marker for predicting cognitive decline in PD. We propose a new framework regarding ‘α-synucleinopathy–glymphatic system–PD’, which may enhance our understanding of the pathophysiology of PD in the prodromal and clinical phases, potentially facilitate earlier diagnosis, and improve the prognostic accuracy of PD. However, the potential of using DTI-ALPS to monitor disease progression or pathological changes related to PD is worth validating. Further development of imaging methods for glymphatic system function is also warranted.

## Methods

### Participants

We performed the study at the Second Affiliated Hospital, Zhejiang University School of Medicine, between January 2017 and February 2021 by using the STROBE cross-sectional reporting guidelines^52^. This study was approved by the medical ethics committee of the Second Affiliated Hospital, Zhejiang University School of Medicine. All participants provided written informed consent before being enrolled. The participants were recruited according to the following criteria: patients with PD were diagnosed according to the criteria of the UK Parkinson Disease Society Brain Bank by at least two experienced neurologists^53^. Participants who met the American Academy of Sleep Medicine criteria for possible iRBD were assigned to the piRBD group [a cut-off score of 17/18 points for the overall scale and 7/8 points for the factor II subscale of the REM Sleep Behaviour Disorder Questionnaire-Hong Kong (RBDQ-HK) scale]^50,54^. All piRBDs were examined by two neurologists to exclude motor signs of parkinsonism. Several community volunteers served as healthy controls (HCs). Participants who met the following criteria were excluded: (1) presence of other causes of PD syndrome, such as metabolic-, immune-, toxicity-related and drug use-related factors; (2) obstructive sleep apnoea hypopnea syndrome, sleep-related epilepsy, night terror/sleepwalking, nocturnal paroxysmal dystonia, secondary RBD caused by drugs, and other sleep disorders that have been associated with dream enactment, including psychiatric, autoimmune and infectious neurological disorders^55^; (3) other neurodegenerative diseases, such as multiple system atrophy, progressive supranuclear palsy and dementia with Lewy bodies; (4) a history of cerebrovascular accident, such as cerebral infarction or cerebral haemorrhage; (5) cerebral structural abnormality, such as brain tumour and trauma; (6) intracranial vein morphology, such as deep vein thrombosis, arteriovenous malformations, and abnormal venous development; (7) severe cognitive dysfunction [Montreal Cognitive Assessment (MoCA) score<10 and/or Mini-Mental State Examination (MMSE) score<9]^56,57^; (8) left- or bilateral-handed; (9) blurred or poor-quality MR images or (10) inability to undergo MRI owing to contraindication and/or refusal to sign an informed consent form. The clinical evaluation and MRI scan were performed on the same day. For patients with PD who were undergoing anti-parkinsonian therapy, clinical evaluation and MRI were performed in the morning after withdrawing from any anti-parkinsonian medications overnight (at least 12 h on ‘drug-off status’) to minimise potential pharmacological effects. During the research period, patients with PD who were followed up over 12 months and agreed to participate in the longitudinal study were invited to undergo longitudinal MRI scanning, which yielded a follow-up group of 50 patients with PD (mean follow-up time interval: 23.36 ± 13.23 months) (Table 2). Figure 1a depicts the flowchart of patient recruitment.

### Baseline variables and clinical assessment

We performed clinical evaluations for all participants. The variables of interest and the assessment methods used included the following:

(i) Demographics: age, sex, education history, symptom onset, disease duration, history of smoking and history of diseases (hypertension, hyperlipidaemia and hyperglycaemia).
(ii) Drug dosage: total daily levodopa equivalent dose (LED).
(iii) Motor symptoms: Unified Parkinson’s Disease Rating Scale-Part III, Hoehn–Yahr scale (H–Y stage).
(iv) Cognition and neuropsychological testing: cognition testing including MMSE and MoCA; neuropsychological features including Hamilton Depression Scale (HAMD) and Hamilton Anxiety Scale (HAMA).
(v) Sleep disturbance^58^: Epworth Sleepiness Score (ESS), Parkinson’s disease sleep scale (PDSS), and RBDQ-HK scale (the RBDQ-HK scale was evaluated in each HC and piRBD, as well as in 88 patients with PD).

### MRI data acquisition and processing

All participants were scanned using a 3.0-Tesla MRI scanner (General Electric Medical Systems, Discovery 750, Boston, MA, USA) equipped with an 8-channel head coil at the Department of Radiology of the Second Affiliated Hospital of the Zhejiang University School of Medicine. During MRI scanning, the head was stabilised using restraining foam pads, and earplugs were provided to reduce the noise. Enhanced susceptibility-weighted angiography images were acquired using a gradient recalled echo sequence with the following parameters: repetition time = 33.7 ms; first echo time/spacing/eighth echo time = 4.556 ms/3.648 ms/30.092 ms; flip angle = 20°; field of view = 240 × 240 mm^2^; matrix = 416 × 384; slice thickness = 2 mm; slice gap = 0 mm; and number of continuous axial slices = 64. DTI images were scanned using a spin echo-echo planar imaging sequence with 30 gradient directions (*b* value = 1000 s/m^2^) and the following parameters: repetition time = 8000 ms; echo time = 80 ms; flip angle = 90°; field of view = 256 × 256; matrix = 128 × 128; slice thickness = 2 mm; slice gap = 0 mm; and number of interleaved axial slices = 67.

#### Quantitative susceptibility mapping data processing

Quantitative susceptibility mapping (QSM) data were processed as described previously^59^. The Susceptibility Tensor Imaging Suite V3.0 software package (University of California at Berkeley, CA, USA, https://people.eecs.berkeley.edu/~chunlei.liu/software.html) was used to calculate the susceptibility maps from the phase images. Specifically, the raw phase was unwrapped using a Laplacian-based phase unwrapping, and the normalised phase was calculated. The normalised background phase was removed using spherical-mean-value filtering (V_SHARP). QSM images were analysed using the STAR-QSM (Streaking Artifact Reduction for QSM) method. The mean signal from each individual brain was used as a susceptibility reference.

#### DTI data processing

DTI images were processed using the FMRIB Software Library (Oxford, UK, FSL, http://www.fmrib.ox.ac.uk/fsl/). The preprocessing procedures included the following steps: (1) brain extraction using FSL’s ‘BET’ tool; (2) eddy-current-induced distortion and head-motion artefact correction using FSL’s ‘eddy_correct’ tool, after which the original b-vectors were rotated according to the affine transformation; (3) diffusion tensor fitting using FSL’s ‘DTIFIT’ tool, in which the colour-coded fractional anisotropy (FA) (Fig. 4a) and diffusivity maps were analysed. We then calculated the diffusivity along the direction of the *x*-, *y*-, and *z* axis (Dx, Dy and Dz, respectively).

#### DTI-ALPS processing

We utilised the method for DTI-ALPS processing and measurement described previously^16–18,60^. Briefly, by first referencing the QSM and colour-FA on coronal scans, we selected a slice at the level of the body of the lateral ventricle in which the medullary veins run perpendicular to the ventricle wall on the axial plane (Fig. 4b). In this area, the PVS is parallel to the medullary vein and is thus mainly in the right–left direction (*x* axis) of the axial plane. This direction is also vertical to the direction of both the projection fibres (mainly in the *z* axis) and association fibres (mainly in the *y* axis) (Fig. 4c, d). Importantly, the subcortical neural fibres pass parallel to the perivascular flow obscuring glymphatic diffusion, and Dy and Dz of the subcortical neural fibres that lay perpendicular to the perivascular flow do not reflect glymphatic diffusion. Thus, the subcortical neural fibre was not calculated in the index. Therefore, in the regions with projection/association fibres, the diffusivity along the x axis is at least partially representative of the diffusivity along the PVS (Fig. 4d). We obtained measurements only in the left hemisphere, as all participants were right-handed, and the fibres were thick to place the region of interest (ROI) on the dominant side^17,18^.

#### ALPS-index calculation

On the colour-coded FA map of the plane at the level of the body of the lateral ventricle, while referencing the QSM, we placed an ROI with a 5-mm diameter in the projection fibres (blue, Fig. 4e) and the area of the association fibres (green, Fig. 4e) in the left hemisphere (since all the participants were right-handed). Two trained neurologists (S. XL and W. ZY) who were blinded to the clinical data evaluated all the images and independently selected the ROI. The selection of ROI by the senior neurologist was used for analysis. Interrater reliability for the segmentation procedure of the ROIs was determined using intraclass correlation coefficients (ICCs).

For each ROI, Dx in the projection (Dxproj) and association (Dxassoc) neural fibre areas on the Dx map, Dy in two fibre areas (Dyproj, Dyassoc) on the Dy map, and Dz in two fibre areas (Dzproj, Dzassoc) on the Dz map were automatically measured to calculate the ALPS index based on the following Eq. (1):

1 $${{{\mathrm{ALPS}}}} - {{{\mathrm{index}}}} = {{{\mathrm{mean}}}}\left( {{{{\mathrm{Dxproj}}}},{{{\mathrm{Dxassoc}}}}} \right)/{{{\mathrm{mean}}}}\left( {{{{\mathrm{Dyproj}}}},{{{\mathrm{Dzassoc}}}}} \right)$$

### Statistical analysis

#### Demographic and clinical differences

The demographic and clinical variables between the groups were analysed using Statistical Product and Service Solutions software (IBM SPSS version 26.0). Statistical plots were generated using GraphPad Prism 8.0a (GraphPad Inc., San Diego, CA, USA). Participants were matched for age and sex by propensity score matching. The results were expressed as the mean ± standard deviation (SD) for continuous variables and as frequencies for categorical variables. Categorical variables were compared using the chi-square test. The normal distribution of data was assessed using the Kolmogorov–Smirnov test. Normally distributed continuous variables were analysed with a two-sample *t* test and analysis of variance, while skewed continuous variables were analysed with the Mann–Whitney *U* test and Kruskal–Wallis test. Furthermore, post hoc analysis was performed, and *P* values < 0.05 (two-tailed) were regarded as statistically significant.

Patients with PD were further separated into several subgroups: (1) according to the H–Y stage (early stage [Early]-PD: H–Y ≤ 2.5 and late stage^61^ -PD: H–Y > 2.5); (2) according to UPDRS III score (mild motor group [UPDRS III < mean – SD], intermediate motor group [UPDRS III between mean ± SD], and severe motor group [UPDRS III > mean + SD]); (3) according to MMSE and MoCA scores (cognitively impaired PD [PD-CI]: MMSE score <27 or/and MoCA score <24; and PD with normal cognition [PDN]: MMSE score ≥27 and MoCA score ≥24)^62^; (4) according to RBDQ-HK score (PD with symptomatic RBD (PD-sRBD): RBDQ-HK score >17 and/or RBDQ-HK factor II > 7, and PD without symptomatic RBD [PD-nRBD]: RBDQ-HK score ≤17 and RBDQ-HK factor II ≤ 7)^50^; (5) according to ESS score (PD with excessive daytime sleepiness [PD-EDS]: ESS score ≥10 and PD without excessive daytime sleepiness [PD-NEDS]: ESS score <10.)^39^ Furthermore, piRBDs were separated into the cognitively impaired piRBDs (piRBD-CI) and piRBDs with normal cognition (piRBDN) groups according to MMSE and MoCA scores. Then, we performed an analysis to explore the difference in the ALPS index between both sexes in the HC group.

#### Correlations between the ALPS index and disease severity

After confirming data normality, Spearman’s correlation analysis was performed to evaluate the relations between the ALPS index and neuropsychological test scores (UPDRS III, RBDQ-HK II, and MoCA) in the piRBD and PD groups. The multiple comparisons for correlation analyses were corrected via false discovery rate (FDR) correction (Benjamini-Hochberg) with adjusted *P* < 0.05.

#### Connections between the ALPS index and progression of PD in the follow-up cohort

Differences of demographics and imaging findings between PD at baseline (PD-BL) and PD at follow-up (PD-F) were analysed with the paired *t* test, Pearson’s chi-square test, or non-parametric tests. The rate of change (%) (Δ/T) was calculated as Eq. (2): [(follow-up − baseline)/time interval (month)]*100^63^. The ALPS index and Δclinical feature (UPDRS III and MoCA)/T were analysed using linear regression analyses. The multiple comparisons for the regression analyses were corrected via FDR correction (Benjamini-Hochberg) with an adjusted *P* < 0.05.

### Reporting summary

Further information on research design is available in the Nature Research Reporting Summary linked to this article.

## Supplementary information

Supplementary File

Reporting Summary

## Data Availability

Clinical and neuroimaging data can be shared on reasonable requests by contacting the corresponding authors.
