# Supplementary material for: Neuroimaging evidence of glymphatic system dysfunction in possible REM sleep behavior disorder and Parkinson’s disease
Source: NPJ Parkinsons Dis. 2022 Apr 29;8:54. doi: 10.1038/s41531-022-00316-9 (PMC9055043; doi:10.1038/s41531-022-00316-9)
Supplement: Supplementary file 1 — Supplementary File [file 41531_2022_316_MOESM1_ESM.pdf]

Supplementary Table 1. Imaging findings of the participant groups

	HC (n=129)	PD (n=168)	piRBDN (MoCA score >24) (n=28)	**p-value**	**Post hoc tests (p-value)**		
				HC vs. PD vs. piRBDN	HC vs. piRBDN	PD vs. piRBDN	HC vs. PD
**ALPS-index**	1.31 ± 0.17	1.20 ± 0.17	1.24 ± 0.16	0.000**	0.040*	0.269	0.000**

	HC (n=129)	PD (n=168)	piRBDN' (MMSE criteria) (n=105)	**p-value**	**Post hoc tests (p-value)**		
				HC vs. PD vs. piRBDN'	HC vs. piRBDN'	PD vs. piRBDN'	HC vs. PD
**ALPS-index**	1.31 ± 0.17	1.20 ± 0.17	1.25 ± 0.16	0.000**	0.003**	0.034*	0.000**

	HC (n=129)	piRBD-CI (n=78)	piRBDN (n=41)	**p-value**	**Post hoc tests (p-value)**		
				HC vs. piRBD-CI vs. piRBDN	HC vs. piRBD-CI	HC vs. piRBDN	piRBD-CI vs. piRBDN
**ALPS-index**	1.31 ± 0.17	1.25 ± 0.17	1.23 ± 0.16	0.004**	0.011*	0.005**	0.475

HC: Healthy control; PD: Parkinson's disease; piRBD-CI: patients with possible idiopathic rapid eye movement sleep behavior disorder with the cognitively impaired; piRBDN: patients with possible idiopathic rapid eye movement sleep behavior disorder with normal cognition; ALPS: analysis along the perivascular space; *Significant result with p <0.05, **Significant result with p <0.01.

Supplementary Table 2. Logistic regression analysis for clinical and imaging discrimination of Parkinson's disease

Variables	Univariate of PD		Multivariable of PD	
	OR (95% CI)	p-value	OR (95% CI)	p-value
Age, years	0.973 (0.947–0.999)	0.044*	0.941 (0.911–0.973)	0.000**
Sex, male	0.632 (0.398–1.003)	0.52	–	–
Education, years	0.930 (0.880–0.983)	0.010*	1.024 (0.946–1.109)	0.559
Smoking	1.056 (0.619–1.802)	0.843	–	–
Hypertension	1.607 (0.972–2.658)	0.065	–	–
Hyperlipidemia	3.730 (1.507–9.228)	0.004**	2.714 (0.954–7.723)	0.061
Hyperglycemia	0.425 (0.174–1.038)	0.060	–	–
Cognitive	2.071 (1.452–2.954)	0.000**	2.473 (1.480–4.132)	0.001**
Mood	3.320 (2.211–4.986)	0.000**	4.250 (2.625–6.881)	0.000**
Sleep	1.041 (0.685–1.582)	0.850	–	–
**ALPS-index**	**0.022 (0.005–0.096)**	**0.000****	**0.014 (0.002–0.075)**	**0.000****

PD: Parkinson's disease; Cognitive: cognitive domain; Mood: mood domain; Sleep: sleep domain; OR: odds ratio; CI: confidence interval;

ALPS: analysis along the perivascular space. *Significant result with $p < 0.05$, **Significant result with $p < 0.01$.

Supplementary Table 3. Corrected and uncorrected values for the correlations and multiple regressions between ALPS-index and various sub-groups

Study	x-axis	y-axis	Group	Statistical analysis	r value	Uncorrected p-value	Corrected p-value
Cross-sectional study	ALPS-index	UPDRS III	PD	Correlation	-0.119	0.126	0.227
			PD-EDS		-0.370	0.019*	0.045*
			PD-NEDS		-0.029	0.740	0.740
			PD-CI		-0.303	0.007**	0.045*
			PDN		0.054	0.614	0.691
			PD-sRBD		-0.431	0.020*	0.045*
			PD-nRBD		0.096	0.471	0.618
		MoCA	PD		-0.055	0.481	0.618
		RBD-HK II	piRBD		-0.236	0.010*	0.045*

Study	x-axis	y-axis	Group	Statistical analysis	r value	Uncorrected p-value	Corrected p-value
Follow-up	ALPS-index (baseline)	UPDRS III /T	PD-FU	Linear regression model (age, sex, and LED as covariates)	B = 0.115	0.442	0.442
		△MoCA/T	PD-FU	Linear regression model (age, sex, education, and LED as covariates)	B = 0.296	0.045*	0.075
		△ALPS-index/T	PD-FU	Linear regression model (age and sex as covariates)	B= -0.496	<0.001**	<0.001**
	△ALPS-index /T	△UPDRS III/T	PD-FU	Linear regression model (age, sex, and LED as covariates)	B = 0.146	0.332	0.415
		△MoCA/T	PD-FU	Linear regression model (age, sex, education, and LED as covariates)	B = -0.300	0.040*	0.075

ALPS, Analysis Along the Perivascular Space; PD: Parkinson's disease; piRBD: patients with possible idiopathic rapid eye movement sleep behavior disorder with normal cognition; PD-EDS: PD with excessive daytime sleepiness; PD-NEDS: PD without excessive daytime sleepiness; PD-CI: cognitively impaired PD; PDN: patients with PD with normal cognition; PD-nRBD: PD without symptomatic RBD; PD-sRBD: PD with symptomatic RBD; PD-FU: PD follow-up; RBDQ-HK II: Rapid Eye Movement Sleep Behavior Disorder Questionnaire-Hong Kong-Part II; UPDRS III: Unified Parkinson's Disease Rating Scale-Part III; MoCA: Montreal Cognitive Assessment; LED: total daily levodopa equivalent dose; △/T: The rate of change (%); *Significant result with p <0.05, **Significant result with p <0.01.

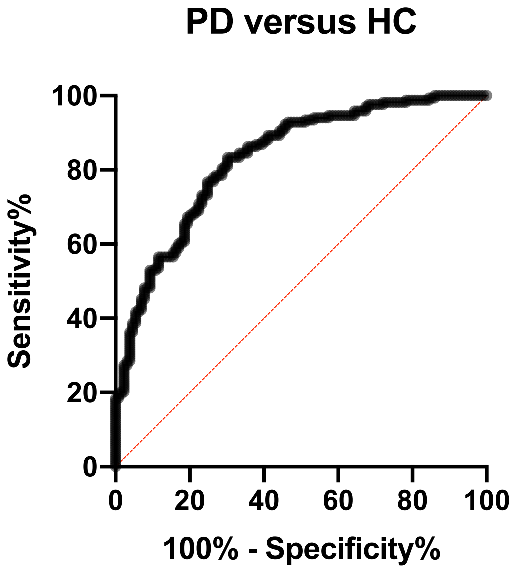

Supplementary Figure 1: Accuracy of the multivariable model for distinguishing PD from HC achieved an AUC of 0.832 (OR = 0.023; 95% CI: 0.786-0.878, p <0.001). Abbreviations: PD: Parkinson’s disease; HC, healthy control; AUC: Area Under Curve.
